# Mechanobiology in Metabolic Dysfunction-Associated Steatotic Liver Disease and Obesity

**DOI:** 10.3390/cimb46070425

**Published:** 2024-07-07

**Authors:** Emily L. Rudolph, LiKang Chin

**Affiliations:** Department of Biomedical Engineering, Widener University, Chester, PA 19013, USA; elrudolph@widener.edu

**Keywords:** mechanobiology, adipocyte biology, hepatocyte biology, intracellular vimentin, extracellular vimentin

## Abstract

With the ongoing obesity epidemic, the prevalence of metabolic dysfunction-associated steatotic liver disease (MASLD) is expected to rise and necessitates a greater understanding of how the disease proceeds from benign excess lipid in hepatocytes to liver fibrosis and eventually to liver cancer. MASLD is caused, at least in part, by hepatocytes’ storage of free fatty acids (FAs) that dysfunctional adipocytes are no longer able to store, and therefore, MASLD is a disease that involves both the liver and adipose tissues. The disease progression is not only facilitated by biochemical signals, but also by mechanical cues such as the increase in stiffness often seen with fibrotic fatty livers. The change in stiffness and accumulation of excess lipid droplets impact the ability of a cell to mechanosense and mechanotranduce, which perpetuates the disease. A mechanosensitive protein that is largely unexplored and could serve as a potential therapeutic target is the intermediate filament vimentin. In this review, we briefly summarize the recent research on hepatocyte and adipocyte mechanobiology and provide a synopsis of studies on the varied, and sometimes contradictory, roles of vimentin. This review is intended to benefit and encourage future studies on hepatocyte and adipocyte mechanobiology in the context of MASLD and obesity.

## 1. Mechanobiology

Historically, biochemical cues were thought to be the primary, if not the only, factor in biological behavior. Within the past several decades, however, the emerging field of mechanobiology has established that this is not the case [1]. A study by biologist D’Arcy Thompson entitled “On Growth and Form”, published in 1917, explains that mechanical forces are reflected in the formation of patterns seen in plants and animals. Since then, new technologies such as atomic force microscopy (AFM) have emerged, paving the way for experiments that connect the physical with the molecular, thereby creating a greater interest in mechanobiology [2].

Mechanobiology is the study of how biological components, including cells, respond to and sense mechanical cues in their environments. These cues consist of applied forces, substrate stiffness, electrical stimulation, and topographical characteristics that may impact cell behavior such as morphology, migration, and proliferation [2,3,4]. Another term of interest is mechanosensing, which describes the ability of a cell to sense physical cues and its microenvironment [2,3]. When a cell transduces external forces into biochemical signals and then responds, this is called mechanotransduction [2,4]. Overall, mechanobiology aims to understand how cells respond to forces and how changes in the mechanical environment are associated with disease [2]. This knowledge can be useful for numerous biomedical applications, for example, tissue engineering, to inform the design of biomaterials with the appropriate mechanical properties [3]. Discoveries resulting from mechanobiology are essential to the field of regenerative medicine and understanding processes in cell regulation and disease [2].

## 2. Mechanosensing from the Extracellular Matrix to the Nucleus

### 2.1. The Extracellular Matrix

The extracellular matrix (ECM) is a crucial component of cellular mechanobiology [3] because of its indispensable role in providing structural support and cell signaling [5]. Consisting of macromolecular proteins such as fibronectin, collagen, and proteoglycans (PGs) [6], the ECM is directly attached to the outside of the cell membrane which, in turn, is attached to cytoskeletal proteins that extend throughout the cell body and to the nucleus [6]. The specific ECM composition depends on the specific tissue [5], but each ECM consists of two primary components—the basement membrane and the interstitial matrix. The basement membrane, composed of proteins including integrins and laminin, is located directly along the cell membrane. The second, the interstitial matrix, is located outside of the basement membrane and includes proteins such as fibronectin and elastin [7].

Mechanical sensing and signaling start when integrins bind to ECM proteins, including PGs, fibronectin, and collagen, which activate the focal adhesion complex. Focal adhesions, comprising the proteins vinculin and focal adhesion kinase (FAK), are responsible for the communication of mechanical cues from the ECM to the cell and vice versa [1].

### 2.2. The Cytoskeleton

The cytoskeleton is attached to the focal adhesions and is composed of microfilaments, microtubules, intermediate filaments (IFs), and cross-linking proteins [8]. The cytoskeleton is largely recognized for its role in the regulation of biochemical cues, cell migration, and cell division [9], although it is essential for mechanosensing and mechanotransduction. Microfilaments are primarily responsible for contraction and motion and have been extensively researched within the field of mechanobiology and disease. Actin is well established as a key mechanosensing protein and is regulated by the myosin–actin–adhesion system or the motor clutch system [10]. Microtubules, which are also well studied in the field of mechanobiology, play a role in transport and cell shape [8]. IFs participate in anchoring organelles, apoptosis, migration, cell adhesion, and cell interactions [8]. IFs are comprised of five alpha-helical rod domains flanked by unstructured head and tail domains that combine to make the IF greater in size than actin but smaller than microtubules, hence the name “intermediate” filament [11].

**Figure 1:**
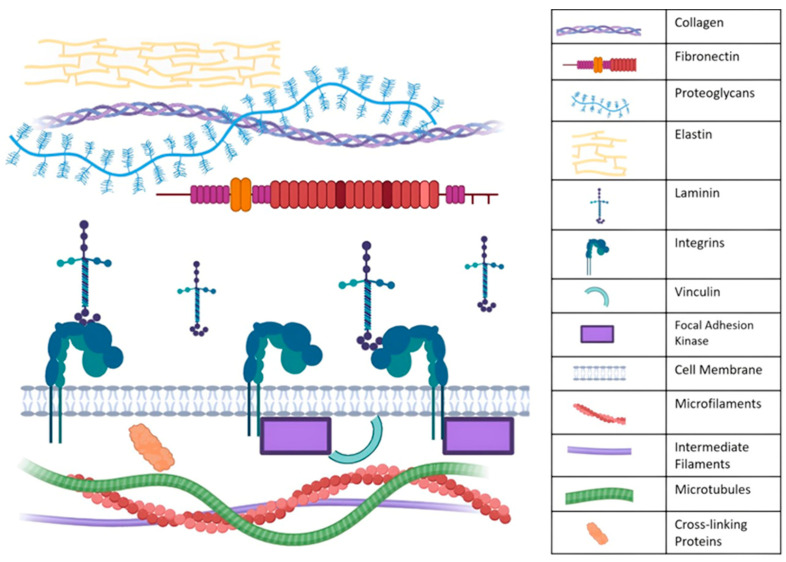
The ECM and key proteins involved in mechanosensing and mechanotransduction. The figure is not drawn to scale and was created with BioRender.com.

All cytoskeletal filaments are semiflexible and display nonlinear elastic behavior, which is specifically demonstrated by actin, vimentin, and neurofilaments [10]. Figure 1 illustrates the ECM and key components involved in mechanosensing as discussed previously.

### 2.3. Novel Roles for the Intermediate Filament Vimentin

The IF protein vimentin has been acknowledged as the shock absorber of the cell because, unlike microtubules and microfilaments which rupture from moderate strains or stress, vimentin can withstand large strains without breaking [12]. When comparing vimentin-null (vim-/-) versus vimentin-expressing wild-type mouse embryonic fibroblasts (mEFs), there are evident differences in the mechanical properties. Under uniaxial compression, vimentin-expressing cells were found to stiffen after each compression cycle, while vim-/- cells spread and softened, underscoring the importance of vimentin in protecting the cell during cyclic compressive loading [11].

In hepatocytes, vimentin is not expressed, except during transformation into cancer cells or during fibrosis. In adipocytes, both in culture and in vivo, vimentin is mostly found intracellularly, forming a cage around the nucleus [13]. During the differentiation of 3T3-L1 cells, vimentin undergoes structural rearrangement. Initially, vimentin appears as a fibrous network throughout the cytoplasm in fibroblastic pre-adipocytes, similar in appearance to mEFs, but then contracts into cage-like structures that are highly associated with the lipid droplets in mature adipocytes. Additionally, vimentin transcription increases [13,14]. Other cytoskeleton proteins, such as actin, tubulin, and cytokeratin, do not exhibit such structures around lipid droplets, so this is a characteristic unique to vimentin in adipocytes [13].

The vimentin network can change under different mechanical conditions. On elastic substrates, vimentin spreads throughout the cell towards the cell membrane. In contrast, vimentin appears in a cage-like manner around the nucleus with less evident filamentous strands throughout the rest of the cell when on viscoelastic substrates, corresponding to a smaller spread area compared to cells on elastic substrates. Similar observations of the vimentin network organization were previously made by Murray and Janmey et al. using human mesenchymal stem cells (hMSCs) seeded onto fibronectin-coated polyacrylamide (PAA) gels of varying stiffness. On soft 200 Pa gels, vimentin surrounded the nucleus like a cage. On stiff substrates of 5 kPa, the cells appeared more elongated, and the vimentin networked extended from the nucleus into 63% of the cytoplasm, not quite reaching the cell periphery. On 30 kPa fibronectin-coated PAA gels, vimentin filled 78% of the cytoplasmic region [12]. On viscoelastic substrates, vimentin-expressing mEFs were significantly more spread and were less likely to form cell clusters than vim-/- mEFs. Vim-/- cells were more likely to be seen in pairs or small clusters. Hence, vimentin facilitated cell–matrix interactions more so than cell–cell interactions [15].

Vimentin that is localized extracellularly, either attached to the cell membrane or secreted outside the cell, has recently been studied, and it was suggested that it has a novel role [11]. Vimentin can be secreted as a response to events, such as stress, senescence, and pathological conditions, as shown in fibroblasts, endothelial cells, and macrophages [11,16]. The role of secreted vimentin in transformed liver cells and adipocytes needs further exploration.

Surface vimentin is one form of extracellular vimentin that remains bound to the surface of the cell [16]. Surface vimentin is used as a receptor for viruses, bacteria, and plasma proteins [17] and is often associated with negative effects, including the facilitation of invading viruses, such as SARS-CoV-2, into the cell [16]. Suprewicz et al. showed via immunostaining that cell surface vimentin is present in the lung and adipose tissues (ATs) as well as sputum, indicating that vimentin has a role in SARS-CoV-2 infection, particularly in these tissues. Additionally, it was shown that purified human vimentin can directly bind to the SARS-CoV-2 pseudovirus and that blocking vimentin with antibodies prevents SARS-CoV-2 infection in human epithelial cell lines [18]. Moreover, others have shown that infection by listeria monocytogenes, foodborne bacteria that can sometimes cross the blood–brain barrier, in human microvascular endothelial cells increases with substrate stiffness and is associated with increased cell surface vimentin and increased FAK activity [19]. It has also been shown that cell surface vimentin increases on infected monocytes during infection by mycobacterium tuberculosis [20] as well as myofibroblasts and activated stellate cells during fibrosis [9].

Secreted vimentin is the second form of extracellular vimentin that is completely released outside of the cell into the ECM and is associated with predominately positive effects. Secreted vimentin can bind to cell receptors, such as insulin-like growth factor 1 receptor, dectin-1, and CD44, to influence cell function [16]. Interestingly, soluble vimentin can be used to prevent infection. It was previously demonstrated that the infection of HEK 293T-hsACE2 cells with the SARS-CoV-2 B.1.1.7 variant with the Wuhan-Hu-1 spike protein could be blocked by 50% when the pseudovirus was first incubated with soluble vimentin [18]. Moreover, others have shown that the pre-treatment of human papillomavirus (HPV) pseudovirus with endogenous vimentin delayed the infection of NIKS cells [16,21]. Secreted vimentin has also been shown to promote the growth of axons, stimulate the tube formation of endothelitranal cells, and have anti-inflammatory effects [9].

The entire repertoire of vimentin functions has not been fully determined. In fact, the literature suggests that the IF has opposing roles. While extracellular vimentin has been shown to have largely favorable effects, several studies have implicated extracellular vimentin in disease processes. For example, macrophages secrete vimentin during atherosclerosis, which stimulates pro-inflammatory cytokines such as interleukin-6 (IL-6) and tumor necrosis factor-α (TNF-α). In addition, secreted vimentin can enhance the migration and phagocytic capabilities of macrophages [9].

## 3. Metabolic Dysfunction-Associated Steatotic Liver Disease and Metabolic Dysfunction-Associated Steatohepatitis

Affecting about 25% of the general population [22], MASLD is an obesity-associated metabolic condition of the liver that is defined by histological diagnosis as the accumulation of lipid in 5% or more of hepatocytes [23,24,25]. MASLD is typically a consequence of AT dysfunction [25,26] and therefore involves both liver cells and adipocytes. The excess free fatty acids (FAs) present in obesity are redirected to the liver when AT reaches its threshold and is unable to store the surplus [27]. As a result, hepatocytes swell with excess lipid uptake, potentially leading to inflammation and liver fibrosis [22,23]. If MASLD goes undetected or treatment does not improve the condition, MASLD can lead to metabolic dysfunction-associated steatohepatitis (MASH) [25]. MASH, present in 2–5% of the population, is a more severe case of MASLD that includes lipotoxicity, which is associated with chronic inflammation. This chronic inflammation can lead to permanent cellular damage. Prolonged MASH can lead to liver fibrosis, and then cirrhosis, and eventually liver cancer [22]. There are limited FDA-approved medications for the direct treatment of MASLD [24], therefore necessitating the need to better understand the disease mechanisms of both hepatocytes and adipocytes.

## 4. The Liver and Hepatocytes

The liver, a red-brown cone-shaped organ located in the abdomen, differentiates toxins and nutrients to properly process them through the body; it also functions to maintain lipid and glucose homeostasis as well as energy balance [24]. The primary cell type of the liver is the hepatocyte [4] which can uptake excess circulating free FAs and store them as triglycerides [28]. It has been established that hepatocytes are mechanosensitive, as shown by changes in morphology, proliferation, and viability due to mechanical stimuli [4,29,30]. The amount and size of the lipid droplets alter how effectively the hepatocyte mechanosenses [23,28,30,31]. Additionally, during liver diseases such as MASLD and MASH, the combination of excess lipid in the hepatocyte and alterations to the extracellular matrix results in changes in the liver’s mechanical properties, which can impair proper liver function [30].

Hepatic stellate cells (HSCs), which make up about 10% of the liver cell population, can accelerate liver fibrosis because of their ability to differentiate into collagen-producing myofibroblasts [12]. In fibrosis models, HSCs and portal fibroblasts represent 95% of collagen-presenting cells, which is indicative of their pivotal roles in the pathogenesis of liver fibrosis. Fibrosis is facilitated by the release of signaling molecules from various cell types that trigger HSCs to transform into myofibroblasts. For example, Kupffer cells release significant amounts of transforming growth factor-beta 1 (TGF-β1), and hepatocytes produce osteopontin and damage-associated molecular patterns. Cholangiocytes recruit fibrogenic cell types and undergo epithelial–mesenchymal transition (EMT), a process known to contribute to liver fibrosis. Additionally, hepatic progenitor cells, bone marrow-derived myofibroblasts, and immune cells are several other cell types that participate in the advancement of liver fibrosis [32,33].

## 5. Mechanobiology in MASLD

Because changes in liver tissue mechanics and mechanical stimuli are likely to be involved in liver disease, there are many studies regarding liver mechanobiology, including MASLD.

The presence of lipid droplets in hepatocytes alter cell elasticity and morphology, as shown in cell culture models of MASLD. Baldini et al. reported that when rat hepatoma FaO cells were fed both fructose and FAs, the lipid droplet size and cell stiffness, measured by single-cell force spectroscopy, were greater than those in the cells treated with fructose, FAs, or TNF-α alone, indicating that fructose and FAs work synergistically to increase steatosis and, subsequently, cell stiffness [34]. Furthermore, when fed FAs alone, the cells became larger and thinner with smooth surfaces. The expression of adipose differentiation-related protein (ADRP) was found to be directly correlated with the size of the lipid droplet. The other protein of interest, IκB kinase β (IKBKB)-interacting protein (IkBip), a marker of liver damage, was directly dependent on the number of lipid droplets. The greater the size of the lipid droplet, the more negatively cell viability was affected. From this, the authors concluded that lipid droplet number alters cell morphology, while lipid droplet size alters cell stiffness.

A study published in 2020 by Chin et al. investigated the effect of lipid droplets on cell morphology, the mechanosensing machinery of hepatocytes, and the mechanosensor yes-associated protein (YAP) [30]. YAP is a transcription factor in the Hippo pathway that is involved in proliferation and cell survival but has also been studied as a sensor for mechanical cues. For example, when cells are on stiff substrates, YAP is in the nucleus, while on soft substrates, it localizes to the cytoplasm. Human hepatocellular carcinoma HuH7 cells and primary human hepatocytes (PHHs) were seeded onto PAA gels of 500 Pa and 10 kPa stiffness, which are representative of normal and cirrhotic liver stiffness, respectively, and glass controls. The cells were then treated with either monounsaturated oleic FA or unsaturated linoleic FA; bovine serum albumin-treated cells served as the control. The oleate-treated HuH7 cells and PHHs stored more lipid than the cells treated with linoleic or controls, and lipid storage was stiff-dependent. The oleate-treated cells stored more lipid on soft gels compared to glass. Increased cell spreading, typically seen on stiff substrates, was reduced with the FA treatment, likely due to the rounding up of the cells. Moreover, the mechanosensing machinery of the cells, including focal adhesions and actin stress fibers, was completely disrupted with the FA treatment, particularly on glass. Interestingly, YAP signal and translocation were not impacted, except when large lipid droplets were induced through insulin treatment. On glass, large lipid droplets were associated with increased nuclear YAP in comparison to small lipid droplets, which correlated to decreased nuclear circularity, thus suggesting that lipid droplet size may impart a pseudo-mechanical signal by deforming the nucleus. This study demonstrates that lipid droplets, particularly when large, can cause hepatocyte dysfunction and suggests a role for the lipid droplet in mechanosignaling.

As shown in hepatocytes [35], HepG2 human hepatoma cells, and HSCs that were all treated with the saturated FA palmitate as well as mouse models of MASLD, vimentin is upregulated and secreted [29,36]. Interestingly, high-fat-diet (HFD) mouse models do not exhibit an increase in vimentin, indicating that vimentin likely has roles in fibrosis and inflammation but does not directly affect lipid accumulation [29].

In hepatocytes, cytokeratin is the most present IF, but vimentin has been shown to increase during EMT and with an increasing cell culture time on tissue culture plastic [37]. In addition to its roles in mechanobiology, cell migration, and proliferation, vimentin is a well-used marker of EMT and is frequently used to label cancer cells. EMT is also involved in HSC activation [37]. The chronic state of inflammation seen in MASLD, particularly in MASH, can induce liver fibrosis and eventually cirrhosis, which are mediated by activated HSCs. Vimentin is essential for HSC transdifferentiation into the myofibroblast, as vimentin knockdown in rat HSCs prevents HSC activation [29].

Guixé-Muntet et al. studied the influence of substrate stiffness on liver cells isolated from normal and carbon tetrachloride-induced cirrhotic rat livers [38]. Hepatocytes isolated from cirrhotic livers that were then seeded onto soft PAA gels showed an improvement in phenotype as measured by the gene expression of hepatocyte nuclear factor 4 α (HNF4α) and albumin as well as urea and albumin secretion. HSCs showed a decrease in α-smooth muscle actin (α-SMA) and collagen. Liver sinusoidal endothelial cells (LSECs) also showed improvement in phenotype. Liver cells isolated from healthy livers exhibited greater nuclear deformation when seeded onto stiff gels compared to when they were seeded onto soft gels. Moreover, all liver cells from cirrhotic livers showed less nuclear deformation on soft versus stiff gels, demonstrating that hepatocytes, HSCs, and LSECs from cirrhotic livers were able to recover their undeformed, spherical nuclear morphology upon being removed from their stiff environments. Mechanical signaling via the cytoskeleton to the nucleus was perturbed by treatment with cytochalasin D and nocodazole to disrupt actin and microtubules, respectively, in all liver cells that were seeded onto stiff substrates. As a result, nuclear deformation decreased due to the uncoupling of the nucleus and the cytoskeleton. Overall, this study demonstrates that a low stiffness environment can recover the functions of hepatocytes, HSCs, and LSECs isolated from stiff fibrotic livers and underscores the importance of mechanical signaling in cell function.

Both the vimentin gene and protein levels have been shown to increase over time in a rat fibrosis model of the liver, mostly in activated HSCs, as reported by Wang et al. [39]. In this study, liver fibrosis was induced in rats using dimethylnitrosamine (DMN) treatment for three days per week for four weeks. Liver injury and severe liver fibrosis were confirmed by routine histology, and immunohistochemistry showed increased vimentin staining in the DMN-treated livers compared to the controls. Next, vimentin was silenced using siRNAs in rat myofibroblast HSC-T7 cells to evaluate the functional role of vimentin in HSC activation. The siRNA was 90% successful in suppressing vimentin and caused a significant increase in peroxisome proliferator-activated receptor γ (PPARγ), a transcription factor that regulates HSC activation; a decrease in proliferating cell nuclear antigen (PCNA); along with decreased amounts of phospho-extracellular signal-regulated kinase 1/2 (p-ERK1/2) and phospho-Ak strain transforming (p-AKT), two proteins involved in HSC activation, in comparison to the controls. When subjected to scratch wound assays, vimentin-silenced HSC-T7 cells migrated at a significantly slower rate than the control cells, correlating to decreases in p-Rac1/cdc42, cdc42, Rac1/2/3, and Rho A/B/C. Additionally, siRNA treatment prevented cell spreading, cell protrusions, and the formation of actin stress fibers in plated HSC-T7 cells. A Western blot analysis showed decreases in filamin A, α-actinin, plectin, talin, and vinculin in comparison to the controls. Hence, vimentin may play a critical role in HSC migration by coordinating cytoskeletal rearrangements. By evaluating the effect of the chemical inhibitors of ERK 1/2, Rho, and AKT on various phosphorylation sites of vimentin, the authors correlated the activation of the IF with HSC proliferation, migration, and transdifferentiation. These results indicate that vimentin plays a critical role in HSC function and activation as well as the arrangement of cytoskeletal components.

Increased levels of vimentin have been measured in the plasma of patients [39] and rats with liver fibrosis in comparison to healthy subjects [29]. Thus, the quantification of vimentin could serve as a potential biomarker or as a diagnostic measure of MASLD and MASH in advanced stages where fibrosis is present. Furthermore, vimentin could serve as a therapeutic target in preventing the onset of fibrosis in MASLD, as more deleterious effects generally follow. A greater understanding of the role of vimentin in MASLD, particularly with respect to HSCs and its interplay with signal transduction pathways, is needed.

## 6. Adipose Tissue and Adipocytes

AT is an endocrine organ composed almost entirely of cells, which are called adipocytes. AT can be divided into two subcategories, white and brown, based on their functions [40]. Both brown and white AT (BAT and WAT, respectively) store FAs. BAT releases this energy for thermogenic purposes, while WAT retains the lipid droplets as triglycerides and is commonly associated with obesity [41]. Subcutaneous white AT (sWAT) and visceral WAT (vWAT) are the two primary fat depots in the body [42]. Visceral fat is wrapped around vital organs, including the liver, while subcutaneous fat is located directly under the skin to act as a mechanical cushion, protection for dermal tissue, and thermal insulation [14]. In this review, only WAT and white adipocytes will be discussed.

Volume, area, and circularity are common metrics used to quantify adipocyte morphology, hyperplasia, and hypertrophy. Hyperplasia refers to a large number of adipocytes that are small in size, while hypertrophy is defined as an increase in cell size but not necessarily in number [43]. Hypertrophy during obesity is associated with glucose intolerance; cell size, particularly of subcutaneous adipocytes, is more greatly increased in type 2 diabetes mellitus (T2DM) [44]. In MASLD, significantly larger adipocytes are present in comparison to those in normal human subjects [45]. The rise in ECM proteins and tissue stiffness from fibrosis, as seen in different metabolic diseases such as obesity and MASLD, limits adipocyte growth [44].

## 7. Mechanobiology in Obesity

Obesity is the accumulation of AT in the body, which can be a precursor to health complications such MASLD [46]. Obesity is marked by an increase in adipocyte size in sWAT and vWAT [42]. Thirty-eight percent of adults are obese or overweight according to the 2023 World Obesity Atlas [47,48]. In 2030, 78% of the adult population is predicted to be overweight or obese [48], greatly increasing the risk for MASLD and potentially liver cancer. Increased caloric intake and decreased physical activity are believed to be primary initiators of this epidemic [47]. At the onset of obesity, adipocytes grow without any increase in angiogenesis, consequently generating a hypoxic (decreased oxygen) milieu. Hypoxia induces ECM deposition, causing gene expression changes that drive the fibrosis and inflammation seen in obesity [49]. In a hypoxic environment, the ACTA2 gene and α-SMA protein are upregulated, resulting in increased actin stress fibers. Interestingly, megakaryoblastic leukemia 1 (MKL1), a mechanosensitive transcription factor, translocates to the nucleus during hypoxia, further promoting stress fiber formation [50]. The accumulation of lipid droplets, either during differentiation or hypertrophy, causes a downregulation and rearrangement of cytoskeletal proteins including vimentin, actin, and tubulin to make space for the growing lipid droplet [13,14]. Hypertrophy has been shown to increase adipocyte stiffness from the range of 300–900 Pa to 2 kPa. Adipocyte cell stiffness may place mechanical constraints on the AT, which also experiences an increase in stiffness [14].

Obesity is a multifaceted disease influenced by various factors, including inflammation and the microenvironment, which function together to progress the disease and promote adipogenesis [51]. Obesity is marked by fibrosis, which is an increase in ECM production. In a study published in 2023, Chen et al. found that COL6A1, a collagen gene, was increased in the vWAT and sWAT of humans with obesity [52]. In mice that were fed HFD, significant increases in COL1A1, COL6A1, along with other collagen genes were observed in inguinal sWAT and perigonadal WAT in comparison to mice that were fed standard chow. The observed increase in ECM production is not only a consequence of obesity but also a cause, as shown in a study which treated 3T3-L1 adipocytes with oleate and/or synthetic polymers that act as macromolecular crowders (MMC) to essentially mimic fibrosis in cell culture. MMC treatment increased the expressions of collagens, laminins, and genes related to adipogenesis to a greater extent than oleate treatment alone in comparison to the untreated controls, and these effects were even more pronounced with the combination treatment involving both oleate and MMC. Cells treated with both oleate and MMC also had the greatest amount of lipid uptake.

Using a HFD mouse animal model to study obesity, Roh et al. analyzed gene expression and proteins including actin and PPARγ [53]. The gene expression of myofibroblast markers, including COL1A1 and ACTA2, significantly increased in the adipocytes of the HFD-fed mice, and these cells exhibited pro-inflammatory and pro-fibrotic phenotypes compared to those that were fed standard chow. PPARγ is a ligand-activated transcription regulator in adipocytes that is essential for adipocyte function, survival, and differentiation. Adipocytes in the HFD environment expressed the downregulation of PPARγ and the activation of the transforming growth factor β-suppressor of mothers against decapentaplegic (TGFβ-SMAD) pathway, which is associated with T2DM and fibrosis. HFD also induced the formation of dense, mesh-like cortical actin just underneath the cell membrane, indicating that complex cellular remodeling occurred. These findings are supported by a study published by Hansson et al., which showed that the adipocytes were larger and had greater actin filament numbers and lengths under HFD conditions compared to standard chow [54]. Additionally, the reversal of adipogenesis was investigated. After two weeks of standard chow after HFD feeding, the adipocytes shrank, and the mice showed similar fat masses and blood glucose levels as the lean controls.

YAP and its paralog transcriptional coactivator with PDZ-binding motif (TAZ) are transcriptional cofactors that regulate cell survival and are activated during obesity [55]. In obesity, YAP/TAZ induces the upregulation of anti-apoptotic factors and the downregulation of pro-apoptotic factors, which stimulate adipocyte survival in an obese environment. Under lean conditions, YAP/TAZ are located in both the cytosol and nucleus of white adipocytes of murine and human subjects, but when fed HFD, YAP/TAZ translocate to the nucleus. Additionally, the gene expressions of YAP/TAZ target genes increased for both the HFD-fed mice and human subjects. When the YAP/TAZ knockout mice were fed a HFD, they gained significantly less weight, presented less vWAT and sWAT, and had improved glucose tolerance in comparison to the HFD-fed wild-type mice. Increased cell death was also noted with YAP/TAZ knockout, concomitant with an upregulation in the pro-apoptotic gene BIM. To understand the mechanism by which YAP/TAZ translocates to the nucleus, 3T3-L1 adipocytes were treated with various cytokines such as IL-1β and TNF-α, which induced the expression of YAP/TAZ target genes and promoted YAP/TAZ nuclear translocation. Interestingly, treatment with various pharmaceuticals to inhibit RhoA, depolymerize actin, and stabilize myosin suppressed YAP/TAZ activation. Inhibition studies suggest that YAP/TAZ nuclear translocation is dependent on the c-Jun N-terminal kinase (JNK) pathway, emphasizing the role of YAP/TAZ in mechanosensitive processes.

The role that YAP plays in adipogenesis is not fully elucidated, but the level of YAP in MSCs is a key indicator of adipo-osteogenesis differentiation [56]. MSCs treated with the reagent dobutamine hydrochloride (DH) showed an increase in phosphorylated, inactive YAP, while cells treated with lysophosphatidic acid (LPA) showed an increase in active YAP. The addition of DH to MSCs, which deactivated YAP, significantly increased intracellular fat droplets in contrast to the controls, while LPA promoted the differentiation of MSCs into osteoblast-like cells, as shown with alizarin red calcium staining.

Several studies have demonstrated that adipocytes are indeed mechanosensing [13,27,49,51,52,53,54,55,56,57,58]. Three-dimensional hydrogels made of collagen without or with ethylene glycol-bis-succinic acid N-hydroxysuccinimide ester (PEGDS) were engineered to create soft and stiff environments, respectively [49]. Adipocytes derived from bone marrow-derived human MSCs (BM-hMSCs) seeded into scaffolds showed different responses depending on stiffness. In the stiff environment, adipocytes formed more well-defined stress fibers with more remodeling than in the soft environment. The quantification of fibrotic genes, including COL1A1, COL6A1, and COL6A3, as well as the enzymatic crosslinker LOX, was increased significantly in the stiff versus soft environment. Interestingly, adiponectin, leptin, and other adipokines had no differences between soft and stiff gels. The profibrotic adipocyte phenotype seen in stiff matrices was reversed upon treatment with Y-27632 to inhibit Rho-associated protein kinase (ROCK). ROCK inhibition resulted in significant decreases in COL1A1, COL6A1, COL6A3, PPARγ, and LOX. These results indicate that actin mediates mechanical cues in the adipocyte.

A paper by Kim et al. demonstrated that vimentin is at least partly responsible for the accumulation of FAs under HFD conditions, leading to a higher likelihood of obesity and insulin resistance [27]. Vimentin knockout mice (vim-/-) were fed a HFD for 10 weeks, and a number of obesity outcomes were measured. HFD-fed vim-/- mice weighed 26% less on average, had smaller epididymal and subcutaneous fat depots, fewer macrophages as determined by F4/80 staining, and improved glucose tolerance, but they had higher serum triglycerides than the HFD-fed wild-type controls. However, the adipocytes of epididymal adipose from HFD-fed vim-/- mice were larger than those from the control mice, but the subcutaneous adipocytes were smaller. Vimentin deletion improved lipid storage in epididymal adipocytes, and this result supports the concept that different fat depots have different roles. Additionally, vim-/- adipocytes expressed 58% less membrane-bound CD36, a protein complex involved in FA transport, and 50% less membrane-bound glucose transporter type 4 (GLUT4), a glucose transporter regulated by insulin, than the control cells. Hence, vimentin is involved in the trafficking of CD36 and GLUT4 to the adipocyte cell membrane. The experimental work by Takahashi et al. also suggested that vimentin is involved in GLUT4 intracellular trafficking, but the results contradict the aforementioned study by Kim [57]. When vimentin was silenced in 3T3-L1 adipocytes using siRNA, there was a significant decrease in the glucose medium levels compared to the untreated controls, indicating an increase in membrane-bound GLUT4. Therefore, more detailed studies are necessary to uncover the detailed mechanism of vimentin-mediated GLUT4 translocation to the cell membrane.

**Figure 2:**
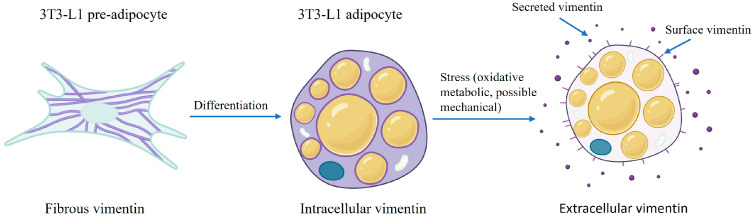
Schematic of intracellular and extracellular (surface and secreted) vimentin after differentiation and stress induction in adipocytes. Purple = vimentin, yellow = lipid droplet, blue = nucleus. Not drawn to scale. Created with BioRender.com accessed on 31 May 2024.

Vimentin alters its structure and location within the cell during the differentiation of fibroblastic 3T3-L1 preadipocytes into adipocytes (Figure 2). The rearrangement of vimentin during differentiation indicates that vimentin has a role in lipid droplet formation, growth, and stabilization. During the early differentiation stage of 3T3-L1 cells, vimentin is filamentous and extends throughout the cytoplasm. During the later stages of adipocyte differentiation, vimentin appears in a cage-like manner around the lipid droplet. As shown by Franke et al., once 80% of 3T3-L1 cells are fully differentiated, all of the vimentin is located at the surface of the many lipid droplets, encaging the lipid droplets in the mature adipocytes [13]. The remaining 20% of cells that were not fully differentiated still exhibited fibrous bundles of vimentin in the cytoplasm. The vimentin amount and lipid droplet size were not directly correlated. Adipocytes isolated from rat epididymal fat pads showed a similar vimentin structure around the lipid droplets.

In a study by Park et al. investigating extracellular vimentin, 3T3-L1 adipocytes were treated with oxidized low-density lipoprotein, a marker of oxidative stress that is associated with obesity, insulin resistance, and metabolic syndrome [58]. Oxidative stress treatment induced the secretion of vimentin after 24 h, as shown by an enzyme-linked immunosorbent assay (ELISA) (schematically shown in Figure 2). When soluble vimentin was added to the media, the adipocyte size and individual lipid droplet size increased. Additionally, glucose and FA uptake increased in the vimentin-treated adipocytes in comparison to the control, likely due to increased GLUT1, GLUT4, and CD36 located in the plasma membrane of the cell. Additionally, the expression of hypoxia-inducible factor 1α (HIF-1α), a key regulator of hypoxic response and a transcription factor for GLUT1, was upregulated, while transcription factors involved in adipogenesis, such as the master regulator PPARγ, decreased with vimentin treatment. Moreover, soluble vimentin induced endoplasmic reticulum stress and decreased autophagy. The authors suggested that extracellular vimentin may act as an adipokine, mediating adipogenesis and energy metabolism under conditions of oxidative stress.

## 8. Concluding Remarks

Excess FA accumulation from caloric overconsumption and decreased physical activity can lead to obesity. When dysfunctional adipocytes are saturated with lipid, free FAs are redirected to hepatocytes in the liver, resulting in MASLD and potentially the more severe case of MASH, as well as other comorbidities.

The role of mechanics in liver diseases, such as fibrosis and cancer, has been well established given that these conditions dramatically change liver tissue stiffness as a result of excessive ECM production. However, the significance of mechanics in fatty liver disease, especially outside the context of fibrosis, has not been extensively studied, although it is a topic of ongoing research, as demonstrated by the numerous papers highlighted in this review article. Research has shown that large amounts of lipid in the cell can result in cell and tissue stiffening and nuclear deformation, which can affect mechanosensitive proteins such as actin and YAP. Oxidative, metabolic, and possibly mechanical stress assist in further progressing disease.

**Figure 3:**
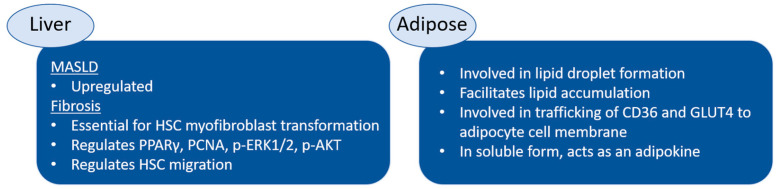
The established roles of vimentin in liver and adipose tissue. The role of vimentin in MASLD and obesity has not been fully elucidated.

A potential novel target that we highlight in this review is the intermediate filament vimentin (Figure 3). Cellular stress is known to induce the secretion of the mechanosensitive IF protein in fibrotic cell types, but limited research has been conducted on the role of vimentin in hepatocytes and adipocytes. Although hepatocytes do not typically express vimentin, adipocytes do, and therefore, vimentin could be involved in mechanosensitive processes that affect the interplay between these two key cell types in MASLD and obesity. Furthering our understanding of the roles of vimentin and its involvement in disease mechanisms could help develop the IF as a possible biomarker for MASLD and obesity and help identify possible solutions.

## Data Availability

Not applicable.
